# Elucidating the Role of THPO and Related Molecular Markers in Lymph Node Metastasis and Prognosis of Gastric Cancer: Insights From TCGA Data Analysis

**DOI:** 10.1155/genr/1438367

**Published:** 2025-09-26

**Authors:** Hong Zhou, Hongbin Liu, Shuyan Liu, Jinfeng Qian

**Affiliations:** Department of Pathology, Affiliated Hospital 2 of Nantong University, Nantong 226001, Jiangsu, China

## Abstract

**Background:** Gastric cancer poses a substantial public health burden, with rising mortality rates in metastatic stages. Elucidating the molecular mechanisms underlying lymph node metastasis is critical for developing novel therapeutic interventions.

**Methods:** Using data from the Cancer Genome Atlas (TCGA), we stratified gastric cancer patients by lymph node metastasis stage (N0–N3) to identify key molecular determinants of metastatic progression. Integrated bioinformatic analyses included differential gene expression profiling, protein–protein interaction networks, survival analysis, and immune microenvironment characterization, with a focused investigation of THPO.

**Results:** We identified metastasis-associated genes, notably THPO, which exhibited stage-dependent upregulation in advanced lymph node metastasis (N3). Elevated THPO expression correlated significantly with adverse prognostic outcomes, including reduced overall survival, disease-free survival, and progression-free survival (all *p* < 0.05). Mechanistically, THPO promoted epithelial–mesenchymal transition and showed a positive correlation with M2 macrophage infiltration, implicating it in tumor progression. Furthermore, a THPO-centric prognostic signature demonstrated high accuracy in predicting 1-, 3-, and 5-year survival rates (AUC > 0.80), supporting its clinical utility. Furthermore, THPO knockdown in MKN-45 cells suppressed migration and blunted the EMT pathway, confirming its prometastatic role in gastric cancer.

**Conclusion:** Our findings establish THPO as a promising biomarker and therapeutic target in gastric cancer. Molecular insights into lymph node metastasis may facilitate the development of precision prognostic tools and tailored therapeutic strategies, highlighting the imperative for further mechanistic and translational studies.

## 1. Introduction

Gastric cancer represents a major global health burden, ranking among the most common malignancies worldwide and exhibiting the second- to third-highest cancer-related mortality rates [1–3]. Early-stage disease, confined to the mucosa or submucosa, demonstrates favorable prognosis with 5-year survival rates reaching 90% [4]. However, survival outcomes plummet dramatically to 10% in metastatic disease and 20%–40% in locally advanced cases with regional metastasis [5, 6].

Recent advances in public health initiatives, population-based screening programs, and endoscopic imaging technologies have significantly elevated the detection of early-stage gastric carcinomas [7]. The implementation of minimally invasive interventions, notably endoscopic submucosal dissection and endoscopic mucosal resection, has substantially improved therapeutic outcomes [8]. These approaches reduce perioperative morbidity, decrease healthcare expenditures, and enhance quality-of-life metrics while maintaining favorable safety profiles. Nevertheless, the persistent risk of occult lymph node (LN) metastasis following these procedures remains a critical clinical concern due to its profound impact on long-term prognosis [9].

The Union for International Cancer Control (UICC) highlights TNM staging, particularly LN classification, as a validated prognostic determinant [10]. Occult LN metastasis poses a significant risk for postoperative recurrence and distant dissemination, supported by evidence of substantial local (15%–25%) and systemic (30%–45%) recurrence rates following local resections [11].

Established clinicopathological predictors of recurrence, including tumor differentiation grade, size, resection margin status, and lymphovascular invasion, guide clinical management. Nevertheless, molecular mechanisms underlying LN metastasis in early gastric cancer remain poorly characterized [12, 13]. This knowledge gap highlights an urgent need for mechanistic studies to optimize surgical decision making and adjuvant strategies following endoscopic resection.

Leveraging the Cancer Genome Atlas (TCGA) dataset, we stratified gastric cancer patients into N0–N3 categories and identified a core set of molecules, including ABCA6, DCLK1, and ADCYAP1, whose expression is tightly linked to LN metastasis. Among these, THPO exhibited progressive upregulation across advanced N stages and was significantly associated with inferior overall survival. Gene set enrichment analysis (GSEA) implicated THPO in the induction of epithelial–mesenchymal transition (EMT). Furthermore, THPO expression correlated positively with tumor-infiltrating M2-polarized macrophages and other immunosuppressive cell subsets. Furthermore, THPO knockdown in MKN-45 cells suppressed migration and blunted the EMT pathway, confirming its prometastatic role in gastric cancer. A THPO-centered prognostic signature demonstrated robust discriminatory power for survival prediction, underscoring its promise as a prognostic biomarker and putative therapeutic target in gastric cancer.

## 2. Methods

### 2.1. Data Acquisition and Patient Selection

Clinical metadata and RNA-sequencing (RNA-seq) profiles of gastric adenocarcinoma (STAD) were retrieved from TCGA via the Genomic Data Commons (GDC) Data Portal [14]. Raw counts and corresponding clinical records were harmonized and subjected to rigorous quality control: samples with incomplete LN staging information, missing RNA-seq data, or ambiguous histology were excluded. Gene-level counts were normalized using the trimmed mean of M-values (TMM) method and log2-transformed for downstream analyses. Patients were stratified into pathologic N categories (N0, N1, N2, and N3) according to the eighth edition of the American Joint Committee on Cancer (AJCC) staging manual. To maximize biological contrast between absence and extensive dissemination, we focused on the two extremes: N0 (*n* = 124, no LN involvement) and N3 (*n* = 82, ≥ 7 positive LNs). This stringent selection scheme minimizes intermediate heterogeneity and accentuates molecular signatures underlying progressive LN metastasis in gastric cancer.

### 2.2. Differential Expression Analysis

Differential gene expression analysis between N0 and N3 stage gastric cancer tissues was conducted using the DESeq2 R package. Genes with an absolute log fold change (|logFC|) greater than 0.5 and a *p* value less than 0.05 were considered significantly differentially expressed genes (DEGs) [15, 16].

### 2.3. Protein–Protein Interaction Network

The PPI network of the identified DEGs was constructed using the STRING database. Only interactions with a confidence score above 0.4 were included. The network visualization was performed using Cytoscape software [17, 18].

### 2.4. Survival Analysis

Univariate Cox proportional hazards regression analysis was utilized to assess the impact of each DEG on survival. Genes identified as risk or protective factors were included in further analyses. Kaplan–Meier (KM) survival curves were generated to evaluate the effect of THPO expression on overall survival, disease-free survival, and progression-free survival, with log-rank tests used to determine statistical significance.

### 2.5. GSEA

GSEA was performed to identify biological pathways enriched among patients with high versus low THPO expression using the Hallmark gene set from the Molecular Signatures Database (MSigDB) [19]. Normalized enrichment scores and nominal *p* values were calculated to assess pathway activation.

### 2.6. Immune Microenvironment Analysis

The composition of the immune microenvironment was quantified using the Xcell, TIMER, QUANTISEQ, MCPCOUNTER, EPIC, and CIBERSORT algorithms [20–24]. Correlations between THPO expression and the abundance of different immune cell types were determined using Spearman's correlation coefficient.

### 2.7. Genomic Feature Analysis

Correlations between THPO expression and genomic features such as tumor mutational burden (TMB), microsatellite instability (MSI), and mRNA-based stemness indices (mRNAsi and EREG-mRNAsi) were evaluated using Spearman's correlation analysis. Mutation data were analyzed to compare the frequency of mutations in key genes between patients with high and low THPO expression.

### 2.8. Construction of Prognostic Model

A prognostic model was developed using variables significantly associated with survival. LASSO regression was employed to select the most relevant prognostic variables from THPO-related molecules. A multivariate Cox regression model was then used to construct the final prognostic model, and a risk score formula was derived. Receiver operating characteristic (ROC) curves were plotted to assess the model's predictive accuracy for 1-, 3-, and 5-year survival.

### 2.9. Cell Culture and siRNA Transfection

Human STAD MKN-45 cells were maintained in RPMI-1640 medium supplemented with 10% fetal bovine serum (FBS; Gibco) and 1% penicillin–streptomycin (Gibco) at 37°C in a humidified atmosphere containing 5% CO_2_. Cells were routinely passaged every 2-3 days using 0.25% trypsin-EDTA (Gibco) to maintain logarithmic growth. MKN-45 cells (3 × 10^5^ per well) were seeded into 6-well plates and cultured overnight to reach 70%–80% confluence. The siRNA targeting human THPO or a non-targeting control siRNA was prepared at 125 nM and delivered with lipo2000 transfection reagent following the manufacturer's protocol. After 6 h, the medium was exchanged for complete RPMI-1640 and the cells were incubated for an additional 48 h before functional assays. Gene silencing (> 70%) was confirmed by quantitative real-time PCR (qRT-PCR) and western blotting.

### 2.10. qRT-PCR

Total RNA was extracted using TRIzol, quantified by NanoDrop, and reverse-transcribed with a commercial cDNA kit. qPCR was performed in triplicate with SYBR Green on a CFX96 system. THPO mRNA levels were normalized to GAPDH and calculated by the 2 ^ (−ΔΔCt) method.

### 2.11. Western Blotting

Cells were lysed in RIPA buffer supplemented with protease and phosphatase inhibitors (1:100). Equal amounts were resolved on 10% SDS-PAGE, transferred to PVDF membranes, blocked with 5% non-fat milk, and probed overnight at 4°C with anti-THPO (1:1000) and anti-GAPDH (1:5000) antibodies. After HRP-conjugated secondary incubation (1:5,000, 1 h), bands were visualized using ECL reagents.

### 2.12. Statistical Analysis

All statistical analyses were performed using R software (Version 4.0 or above). A *p* value less than 0.05 was considered statistically significant in all analyses unless stated otherwise.

## 3. Results

### 3.1. Identification of Molecules Involved in LN Metastasis in Gastric Cancer

**Figure 1:**
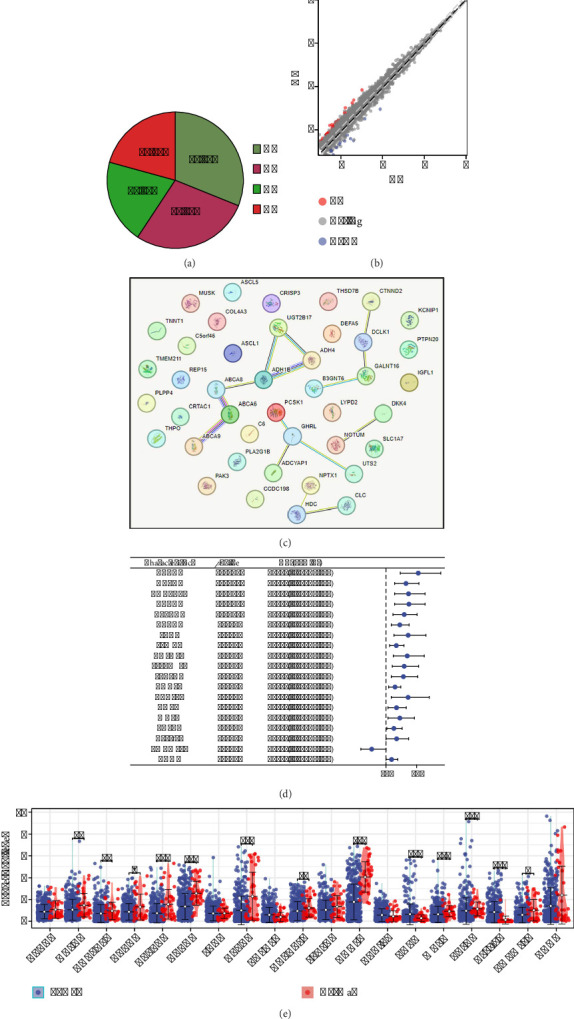
Overview of gastric cancer patient data analysis and molecular identification. (a) Staging of gastric cancer patients from TCGA data, categorizing patients into N0, N1, N2, and N3 stages based on lymph node involvement. (b) Differential gene expression analysis between N0 and N3 stage gastric cancer patients, highlighting genes with a log fold change (|logFC|) greater than 0.5 and *p* value less than 0.05. (c) Protein–protein interaction network of DEGs identified between N0 and N3 stages, illustrating potential interactions and functional associations between these proteins. (d) Univariate Cox analysis of identified DEGs. (e) The expression level of DEGs between normal and gastric cancer tissue.

Firstly, clinical information sourced from TCGA allowed us to categorize gastric cancer patients into N0, N1, N2, and N3 stages (Figure 1(a)). To pinpoint molecules linked to LN metastasis, we analyzed patients at N0 (124 individuals) and N3 (82 individuals) stages for DEGs with significant cutoffs (|logFC| > 0.5 and *p* value < 0.05) (Figure 1(b) and Supporting file 1). The protein–protein interaction network of these DEGs is displayed in Figure 1(c). Univariate Cox analysis identified several DEGs such as ABCA6, DCLK1, and ADCYAP1 as risk factors, while TMEM211 was protective (Figure 1(d)). Furthermore, we assessed the expression differences of these molecules between normal and gastric cancer tissues. It was found that molecules like DCLK1 and ADCYAP1 were downregulated, whereas THPO and C5orf46 were upregulated in gastric cancer (Figure 1(e)). However, we found that C5orf46 was downregulated in N3 patients, so we chose THPO for further analysis (Supporting file 1).

### 3.2. Clinical Role of THPO in Gastric Cancer

**Figure 2:**
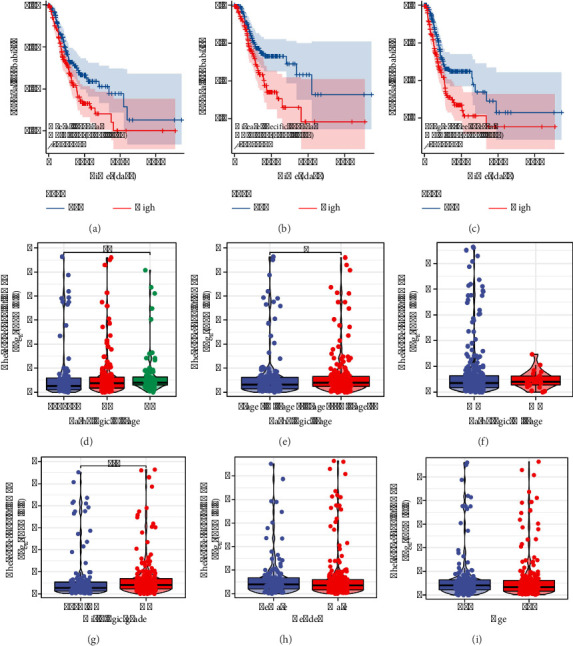
Analysis of THPO expression and its impact on survival in gastric cancer patients. (a) KM survival curves showing the impact of THPO expression on overall survival. (b) KM survival curves for disease-free survival. (c) KM survival curves for progression-free survival. (d) The expression level of THPO in gastric patients with different T stages. (e) The expression level of THPO in gastric patients with different clinical stages. (f) The expression level of THPO in gastric patients with different M stages. (g) The expression level of THPO in gastric patients with histologic grade. (h) The expression level of THPO in male and female gastric patients. (i) The expression level of THPO in gastric patients with different ages.

THPO, found to be upregulated in gastric cancer, was associated with advanced N stages. Higher THPO expression was linked to poorer survival outcomes as shown by KM survival curves (Figures 2(a), 2(b), and 2(c)). Meanwhile, we found that THPO is associated with advanced T stage, clinical stage, and historical grade (Figures 2(d), 2(e), 2(f), 2(g), 2(h), and 2(i)).

### 3.3. Biological Enrichment of THPO in Gastric Cancer

**Figure 3:**
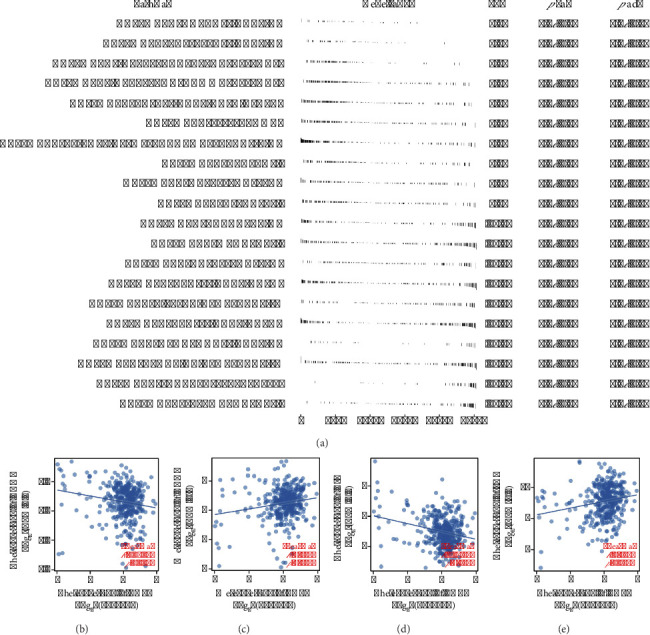
Gene set enrichment and correlation analysis related to THPO in gastric cancer. (a) GSEA showing the upregulation of pathways such as Notch, TGF-β, and interferon responses in patients with high THPO expression. (b–e) Correlation plots illustrating the relationships between THPO expression and key markers of EMT, including negative correlations with CDH1 and VIM and positive correlations with CDH2 and FN1.

GSEA analysis revealed upregulation in pathways such as Notch, TGF-β, and both interferon responses in patients with high THPO expression, pointing to its role in EMT, a critical process in tumor metastasis (Figure 3(a)). Additionally, THPO showed a negative correlation with CDH1 and VIM and a positive correlation with CDH2 and FN1, supporting its role in EMT (Figures 3(b), 3(c), 3(d), and 3(e)).

### 3.4. Role of THPO in the Gastric Cancer Immune Microenvironment

**Figure 4:**
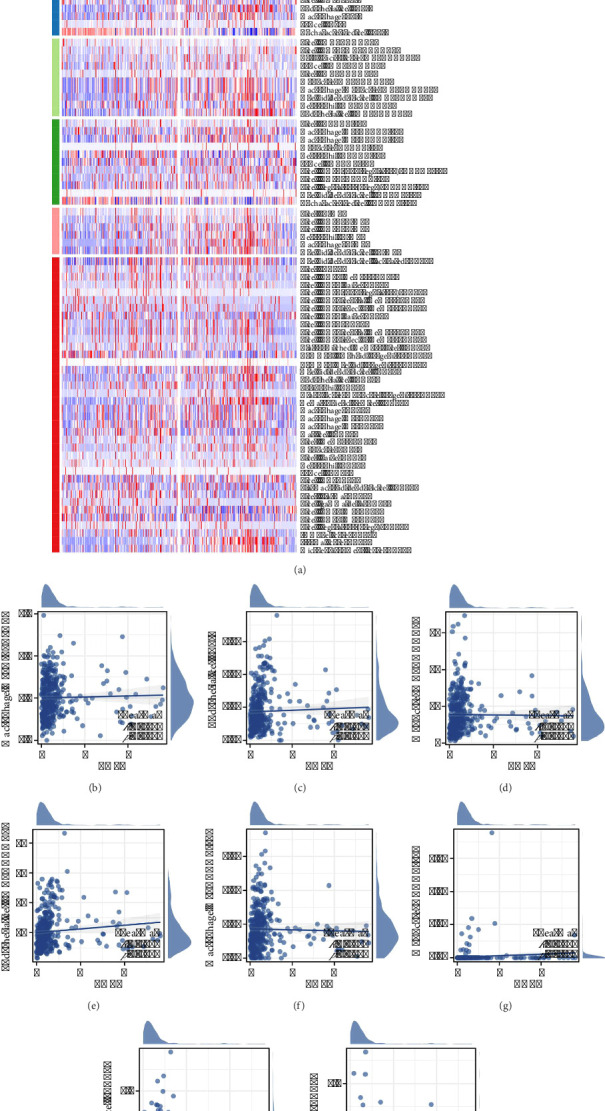
Immune microenvironment analysis in gastric cancer influenced by THPO expression. (a) Overview of the immune microenvironment analysis evaluating the influence of THPO expression. (b–i) Detailed analysis of THPO's correlation with the presence of M2 macrophages, endothelial cells, and monocytes, supporting its role in modulating the tumor immune microenvironment.

The immune microenvironment, crucial for tumor progression, was evaluated using multiple algorithms (Figure 4(a)). THPO showed a positive correlation with the presence of M2 macrophages, endothelial cells, and monocytes (Figures 4(b), 4(c), 4(d), 4(e), 4(f), 4(g), 4(h), and 4(i)).

### 3.5. Genomic Features of THPO in Gastric Cancer

**Figure 5:**
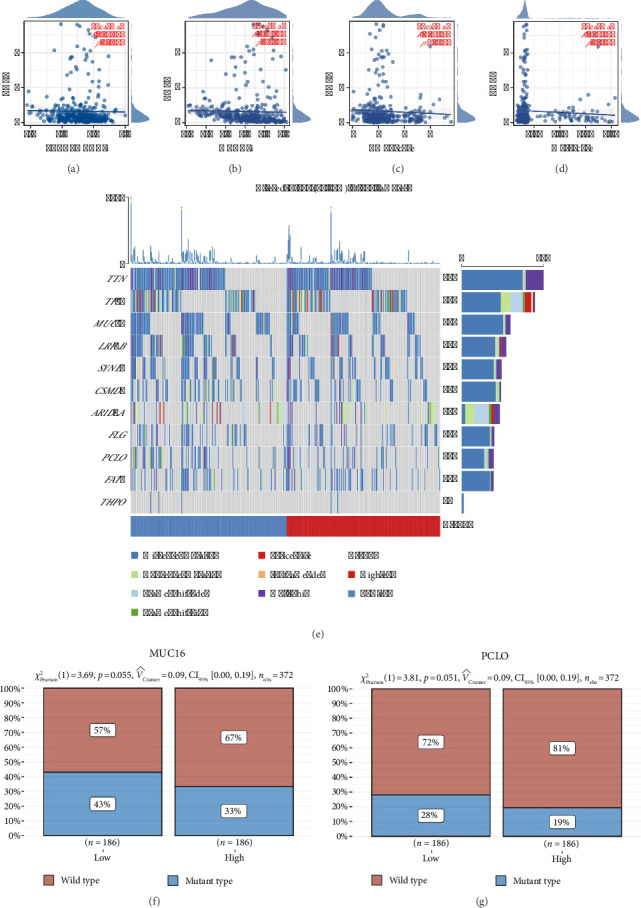
Investigation of the relationship between THPO expression and genomic features in gastric cancer. (a, b) Analysis of the relationship between THPO expression and tumor stemness indices—mRNAsi and EREG-mRNAsi. (c, d) Analysis of the lack of significant correlation between THPO expression and TMB and MSI. (e–g) Mutation analysis in gastric cancer, showing higher mutation frequencies in MUC16 and PCLO in patients with low THPO expression.

We also explored the relationship between THPO and genomic features. THPO was inversely related to tumor stemness indices—mRNAsi and EREG-mRNAsi (Figures 5(a) and 5(b)). However, no significant correlation was found with TMB and MSI scores (Figures 5(c) and 5(d)). Analysis of mutation differences revealed higher mutations in MUC16 and PCLO in patients with low THPO expression (Figures 5(e), 5(f), and 5(g)).

### 3.6. Construction of a Prognostic Model Based on THPO-Related Molecules

**Figure 6:**
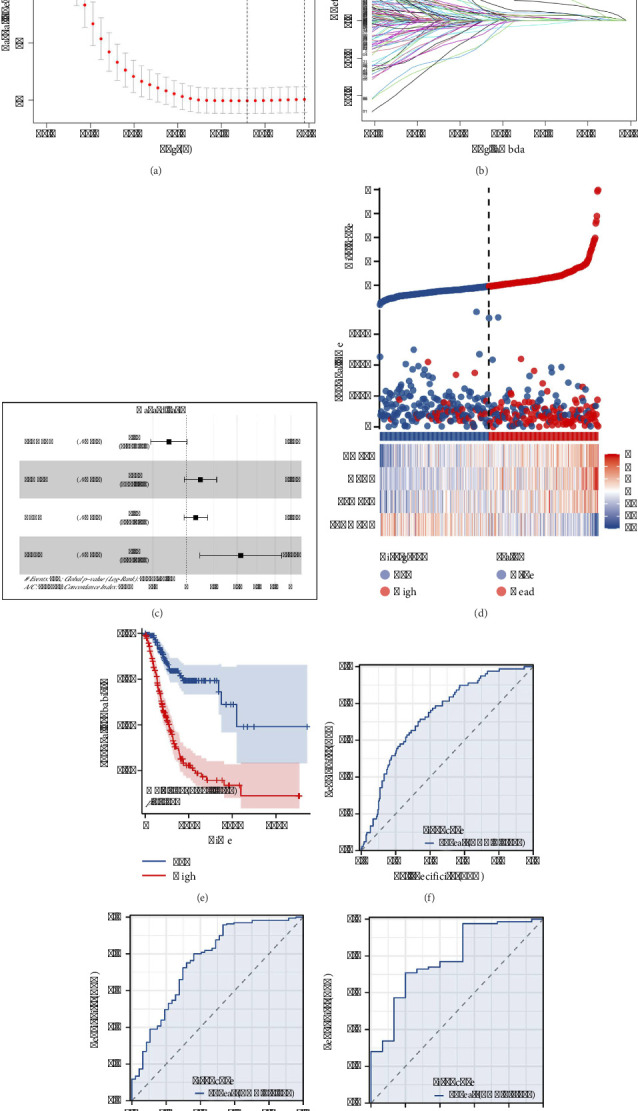
Construction and validation of a prognostic model based on THPO-related molecules. (a, b) Univariate Cox analysis and LASSO regression used to refine prognostic variables based on THPO-related molecules. (c) Multivariate Cox regression model incorporating factors like ZNF22, GPC3, FKBP10, and SLCO4A1 to predict patient survival. (d) Comparison of survival rates between patients classified under high-risk and low-risk categories based on the developed risk score formula. (e–h) Kaplan–Meier and receiver operating characteristic (ROC) curves demonstrating the efficacy of the prognostic model in predicting 1-, 3-, and 5-year survival rates in gastric cancer patients.

Through correlation analysis, significant THPO-related molecules were identified (Supporting file 2). Univariate Cox analysis and LASSO regression helped refine prognostic variables (Figures 6(a) and 6(b)), leading to a multivariate Cox regression model involving ZNF22, GPC3, FKBP10, and SLCO4A1 (Figure 6(c)). The risk score formula developed was “Risk score = SLCO4A1 ∗ −0.117 + FKBP10 ∗ 0.092 + GPC3 ∗ 0.061 + ZNF22 ∗ 0.361.” Patients classified under the high-risk category exhibited a higher mortality rate (Figure 6(d)). KM and ROC curves demonstrated that this model effectively predicts 1-, 3-, and 5-year survival rates in gastric cancer patients (Figures 6(e), 6(f), 6(g), and 6(h)).

### 3.7. THPO Promotes the Migration Ability and EMT Pathway in Gastric Cancer Cell

**Figure 7:**
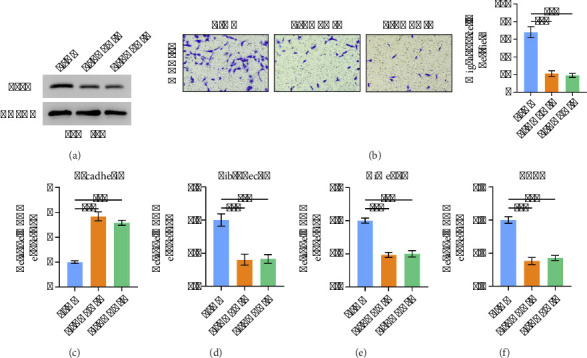
Functional validation of THPO in gastric cancer cell migration and EMT pathway. (a) Western blot confirming the knockdown efficiency of THPO in MKN-45 cells after transient transfection with siRNA targeting THPO. (b) Transwell migration assay demonstrating that THPO knockdown significantly reduces the migratory capacity of MKN-45 cells. (c–f) Results of qPCR reveal that THPO knockdown leads to a marked decrease in the expression of key EMT-related genes.

Subsequently, we investigated the biological role of THPO in the gastric cancer cell line MKN-45. Firstly, we performed transient knockdown of THPO using siRNA and validated the efficiency using western blot (Figure 7(a)). Subsequently, we used transwell experiments to detect changes in the migration ability of gastric cancer cells after knocking down THPO. The results showed that knocking down THPO significantly reduced the migration ability of MKN-45 cells (Figure 7(b)). Our GSEA results indicate a positive correlation between THPO and the EMT pathway. For this purpose, we examined whether knocking down THPO would affect indicators related to the EMT pathway. As expected, knocking down THPO significantly reduced the activity of the EMT pathway (Figures 7(c), 7(d), 7(e), and 7(f)).

## 4. Discussion

Early-stage gastric tumors typically exhibit a lower incidence of LN metastasis compared to advanced disease, attributable to their smaller tumor burden, higher degree of differentiation, and reduced neural or vascular invasion [1, 2, 5]. Endoscopic resection for gastric lesions is indicated primarily for early cancers or precancerous lesions without evidence of LN metastasis [25, 26]. Current clinical assessment of nodal status prior to endoscopic resection relies heavily on imaging modalities such as contrast-enhanced CT, MRI, and endoscopic ultrasound [27]. While these techniques offer distinct advantages for evaluating nodal involvement, their diagnostic accuracy is limited by challenges including inflammatory lymphadenopathy and the difficulty in detecting early metastatic deposits [28]. Critically, validated molecular biomarkers for predicting LN metastasis in early gastric cancer remain elusive [29]. Elucidating the molecular mechanisms underlying lymphatic spread in early-stage disease holds significant promise for refining prognostic stratification and enabling personalized patient management strategies.

In our study, we categorized gastric cancer patients into N0, N1, N2, and N3 stages using clinical information from TCGA and identified key molecules associated with LN metastasis by comparing gene expression between patients at N0 and N3 stages. We found that molecules such as ABCA6, DCLK1, and ADCYAP1 were linked to higher risk, with DCLK1 and ADCYAP1 being downregulated, while THPO and C5orf46 were upregulated in gastric cancer tissues. Notably, elevated THPO expression was associated with advanced N stages and correlated with poorer survival outcomes including reduced overall, disease-free, and progression-free survival. GSEA further revealed that THPO plays a crucial role in pathways promoting EMT, a key process in tumor metastasis [30]. Analysis of the immune microenvironment showed a positive correlation between THPO and the presence of M2 macrophages, endothelial cells, and monocytes. A prognostic model constructed based on THPO and related molecules effectively predicted 1-, 3-, and 5-year survival rates for gastric cancer patients. Furthermore, THPO knockdown in MKN-45 cells suppressed migration and blunted the EMT pathway, confirming its prometastatic role in gastric cancer. These findings not only highlight the significant role of THPO in the progression of gastric cancer but also offer potential biomarkers and therapeutic targets for future treatments.

THPO is primarily known for its role in hematopoiesis, particularly in stimulating the production and maturation of megakaryocytes into platelets in the bone marrow [31]. However, recent studies have extended our understanding of THPO's function beyond hematopoiesis, suggesting its involvement in various malignancies [32]. In gastric cancer, we found that THPO was upregulated in tumor tissues, correlating with advanced disease stages and poorer prognostic outcomes. This association indicates that THPO might influence tumor growth and metastasis, potentially by modulating cellular mechanisms such as cell proliferation, apoptosis, and migration. The study of THPO in the context of cancer provides a compelling example of how molecules known for their roles in normal physiological processes may have critical implications in tumorigenesis and cancer progression.

GSEA has revealed that THPO is involved in activating several key signaling pathways implicated in cancer pathophysiology, notably the Notch and TGF-β pathways, as well as pathways related to interferon responses. These pathways are crucial for the process of EMT, which is a pivotal mechanism in cancer metastasis [33]. EMT endows cancer cells with the ability to dissociate from the primary tumor mass, invade surrounding tissues, and establish secondary tumors, thus facilitating metastasis [34]. The upregulation of THPO and its association with these pathways suggest that it may serve as a regulatory factor in the complex network of signals that promote EMT and tumor progression. Understanding these interactions can provide insights into the molecular underpinnings of gastric cancer and open up potential avenues for targeted therapy.

The interaction between THPO and the tumor immune microenvironment, particularly its association with M2 macrophages, underscores a critical aspect of its role in cancer biology. M2 macrophages, often referred to as tumor-associated macrophages (TAMs), are known for their protumorigenic activities, including support of tumor growth, promotion of angiogenesis, and suppression of antitumor immunity [35]. THPO's positive correlation with M2 macrophages in gastric cancer suggests that it may influence the recruitment or differentiation of these cells, thereby contributing to an immunosuppressive microenvironment favorable for tumor progression. This relationship highlights the dual role of THPO in both promoting tumorigenic pathways and modulating the immune landscape, making it a potentially valuable target for therapies aimed at altering the immune response to gastric cancer.

While our study highlights the significant role of THPO in gastric cancer progression and its association with poor prognosis, several limitations must be acknowledged. First, the entire analysis is based on retrospective data from TCGA, which may harbor selection, collection, and annotation biases. Second, although we observed correlations between THPO expression and M2-like macrophage infiltration, the direct immunomodulatory effects of THPO on the tumor microenvironment require mechanistic validation through dedicated functional assays. Third, and most importantly, the prognostic model built from THPO-related molecules currently lacks both internal and external clinical validation. Therefore, prospective, multicenter studies are essential to confirm the model's accuracy and generalizability before any clinical application.

## Data Availability

Based on reasonable requirements, all data can be obtained from the corresponding author.

## Supporting Information

Supporting Information 1 Supporting file 1. The identified DEGs between N0 and N3 gastric cancer patients.

Supporting Information 2 Supporting file 2. The THPO-related molecules identified by the correlation analysis.

## References

[1] Smyth E. C., Nilsson M., Grabsch H. I., van Grieken N. C., Lordick F. (2020). Gastric Cancer. *The Lancet*.

[2] Karimi P., Islami F., Anandasabapathy S., Freedman N. D., Kamangar F. (2014). Gastric Cancer: Descriptive Epidemiology, Risk Factors, Screening, and Prevention. *Cancer Epidemiology, Biomarkers & Prevention*.

[3] Chandarana C. V., Mithani N. T., Singh D. V., Kikani U. B. (2024). Vibrational Spectrophotometry: A Comprehensive Review on the Diagnosis of Gastric and Liver Cancer. *Current Pharmaceutical Analysis*.

[4] Zhang P., Yang M., Zhang Y. (2019). Dissecting the Single-Cell Transcriptome Network Underlying Gastric Premalignant Lesions and Early Gastric Cancer. *Cell Reports*.

[5] López M. J., Carbajal J., Alfaro A. L. (2023). Characteristics of Gastric Cancer Around the World. *Critical Reviews in Oncology*.

[6] Wang Y., Chen S., Yu P. (2023). SR-BI Expression Regulates the Gastric Cancer Tumor Immune Microenvironment and is Associated With Poor Prognosis. *Biocell*.

[7] Pinsky P. F. (2015). Principles of Cancer Screening. *Surgical Clinics of North America*.

[8] García-Flórez L. J., Otero-Díez J. L. (2015). Local Excision by Transanal Endoscopic Surgery. *World Journal of Gastroenterology*.

[9] Qian Y., Zhai E., Chen S. (2022). Single-Cell RNA-Seq Dissecting Heterogeneity of Tumor Cells and Comprehensive Dynamics in Tumor Microenvironment During Lymph Nodes Metastasis in Gastric Cancer. *International Journal of Cancer*.

[10] Bertero L., Massa F., Metovic J. (2018). Eighth Edition of the UICC Classification of Malignant Tumours: An Overview of the Changes in the Pathological TNM Classification Criteria-What Has Changed and Why?. *Virchows Archiv*.

[11] Zhang C., Xie M., Zhang Y. (2022). Determination of Survival of Gastric Cancer Patients With Distant Lymph Node Metastasis Using Prealbumin Level and Prothrombin Time: Contour Plots Based on Random Survival Forest Algorithm on High-Dimensionality Clinical and Laboratory Datasets. *Journal of Gastric Cancer*.

[12] Huang J., Lucero-Prisno D. E., Zhang L. (2023). Updated Epidemiology of Gastrointestinal Cancers in East Asia. *Nature Reviews Gastroenterology & Hepatology*.

[13] Staderini F., Barbato G., Bottari A. (2023). Effects of the Learning Curve on Operative Time and Lymph Node Harvesting During Robotic Gastrectomy. *International Journal of Medical Robotics and Computer Assisted Surgery*.

[14] Tomczak K., Czerwińska P., Wiznerowicz M. (2015). The Cancer Genome Atlas (TCGA): An Immeasurable Source of Knowledge. *Contemporary Oncology*.

[15] Ritchie M. E., Phipson B., Wu D. (2015). Limma Powers Differential Expression Analyses for RNA-Sequencing and Microarray Studies. *Nucleic Acids Research*.

[16] Yan S., Han Z., Wang T. (2024). Exploring the Immune-Related Molecular Mechanisms Underlying the Comorbidity of Temporal Lobe Epilepsy and Major Depressive Disorder Through Integrated Data Set Analysis. *Current Molecular Pharmacology*.

[17] Shannon P., Markiel A., Ozier O. (2003). Cytoscape: A Software Environment for Integrated Models of Biomolecular Interaction Networks. *Genome Research*.

[18] Guo Y., Liu X. U., Xu Q. I. (2024). Revealing the Role of Honokiol in Human Glioma Cells by RNA-Seq Analysis. *Biocell*.

[19] Subramanian A., Tamayo P., Mootha V. K. (2005). Gene Set Enrichment Analysis: A Knowledge-Based Approach for Interpreting Genome-Wide Expression Profiles. *Proceedings of the National Academy of Sciences*.

[20] Aran D., Hu Z., Butte A. J. (2017). xCell: Digitally Portraying the Tissue Cellular Heterogeneity Landscape. *Genome Biology*.

[21] Li T., Fan J., Wang B. (2017). TIMER: A Web Server for Comprehensive Analysis of Tumor-Infiltrating Immune Cells. *Cancer Research*.

[22] Plattner C., Finotello F., Rieder D. (2020). Deconvoluting Tumor-Infiltrating Immune Cells From RNA-Seq Data Using quanTIseq. *Methods in Enzymology*.

[23] Racle J., Gfeller D. (2020). EPIC: A Tool to Estimate the Proportions of Different Cell Types From Bulk Gene Expression Data. *Methods in Molecular Biology*.

[24] Chen B., Khodadoust M. S., Liu C. L., Newman A. M., Alizadeh A. A. (2018). Profiling Tumor Infiltrating Immune Cells With CIBERSORT. *Methods in Molecular Biology*.

[25] Gotoda T., Ono H. (2022). Stomach: Endoscopic Resection for Early Gastric Cancer. *Digestive Endoscopy*.

[26] Kim G. H., Jung H. Y. (2021). Endoscopic Resection of Gastric Cancer. *Gastrointestinal Endoscopy Clinics of North America*.

[27] Zhang Y., Yu J. (2020). The Role of MRI in the Diagnosis and Treatment of Gastric Cancer. *Diagnostic and Interventional Radiology*.

[28] Xu Q., Sun Z., Li X. (2021). Advanced Gastric Cancer: CT Radiomics Prediction and Early Detection of Downstaging With Neoadjuvant Chemotherapy. *European Radiology*.

[29] Dong D., Fang M. J., Tang L. (2020). Deep Learning Radiomic Nomogram Can Predict the Number of Lymph Node Metastasis in Locally Advanced Gastric Cancer: An International Multicenter Study. *Annals of Oncology*.

[30] Pastushenko I., Blanpain C. (2019). EMT Transition States During Tumor Progression and Metastasis. *Trends in Cell Biology*.

[31] Dasouki M., Saadi I., Ahmed S. O. (2015). THPO-MPL Pathway and Bone Marrow Failure. *Hematology/Oncology and Stem Cell Therapy*.

[32] Shirai T., Revenko A. S., Tibbitts J. (2019). Hepatic Thrombopoietin Gene Silencing Reduces Platelet Count and Breast Cancer Progression in Transgenic MMTV-PyMT Mice. *Blood Advances*.

[33] Kiri S., Ryba T. (2024). Cancer, Metastasis, and the Epigenome. *Molecular Cancer*.

[34] Bakir B., Chiarella A. M., Pitarresi J. R., Rustgi A. K. (2020). EMT, MET, Plasticity, and Tumor Metastasis. *Trends in Cell Biology*.

[35] Yunna C., Mengru H., Lei W., Weidong C. (2020). Macrophage M1/M2 Polarization. *European Journal of Pharmacology*.

